# Sepsis-induced cardiogenic shock: controversies and evidence gaps in diagnosis and management

**DOI:** 10.1186/s40560-024-00770-y

**Published:** 2025-01-02

**Authors:** Ryota Sato, Daisuke Hasegawa, Stephanie Guo, Abdulelah E. Nuqali, Jesus E. Pino Moreno

**Affiliations:** 1 Division of Critical Care Medicine, Department of Medicine, The Queen’s Medical Center, Honolulu, HI USA; 2 Division of Pulmonary, Allergy, and Critical Care, Perelman School of Medicine at the University of Pennsylvania, Philadelphia, PA USA; 3 Division of Pulmonary and Critical Care Medicine, Department of Medicine, The Queen’s Medical Center, Honolulu, HI USA; 4 Department of Medicine, John A. Burns School of Medicine, University of Hawai’i, Honolulu, HI USA; 5 Queen’s Heart Institute, The Queen’s Medical Center, Honolulu, HI USA

**Keywords:** Septic shock, Cardiogenic shock, Sepsis-induced cardiomyopathy, Septic cardiomyopathy, Mixed shock, Mechanical circulatory support

## Abstract

Sepsis often leads to vasoplegia and a hyperdynamic cardiac state, with treatment focused on restoring vascular tone. However, sepsis can also cause reversible myocardial dysfunction, particularly in the elderly with pre-existing heart conditions. The Surviving Sepsis Campaign Guidelines recommend using dobutamine with norepinephrine or epinephrine alone for patients with septic shock with cardiac dysfunction and persistent hypoperfusion despite adequate fluid resuscitation and stable blood pressure. However, the definition of cardiac dysfunction and hypoperfusion in these guidelines remains controversial, leading to varied clinical interpretations. Cardiac dysfunction with persistent hypoperfusion despite restoring adequate preload and afterload is often considered a cardiogenic shock. Therefore, sepsis complicated by new-onset myocardial dysfunction or worsening of underlying myocardial dysfunction due to sepsis-induced cardiomyopathy, resulting in cardiogenic shock, can be defined as “Sepsis-induced cardiogenic shock (SICS)”. SICS is known to be associated with significantly higher mortality. A history of cardiac dysfunction is a strong predictor of SICS, highlighting the need for precise diagnosis and management given the aging population and rising cardiovascular disease prevalence. Therefore, SICS might benefit from early invasive hemodynamic monitoring with a pulmonary artery catheter (PAC), unlike those with septic shock alone. While routine PAC monitoring for all septic patients is impractical, echocardiography could be a useful screening tool for high-risk individuals. If echocardiography indicates cardiogenic shock, PAC might be warranted for continuous monitoring. The role of inotropes in SICS remains uncertain. Mechanical circulatory support (MCS) might be considered for severe cases, as high-dose vasopressors and inotropes are associated with worse outcomes. Correct patient selection is the key to improving outcomes with MCS. Engaging a cardiogenic shock team for a multidisciplinary approach can be beneficial. In summary, addressing the evidence gaps in SICS diagnosis and management is crucial. Echocardiography for screening, advanced monitoring with PAC, and careful patient selection for MCS are important for optimal patient care.

## Introduction

Sepsis is defined as life-threatening organ dysfunction resulting from a dysregulated immune response to infection. Organ dysfunction is indicated by an increase of 2 points or more in the Sequential Organ Failure Assessment (SOFA) score [1]. In addition, septic shock is defined as sepsis requiring vasopressor supports to maintain a mean arterial pressure of 65 mmHg and a serum lactate level greater than 2 mmol/L [1]. Sepsis is characterized by vasoplegia and a hyperdynamic cardiac state, with treatments primarily aimed at restoring vascular tone. In contrast, cardiogenic shock is characterized by low cardiac outputs, high left ventricular filling pressure, high systemic vascular resistance, and decreased perfusion, often defined as hypotension accompanied by evidence of end-organ damage from low cardiac output, with a cardiac index ≤ 2.2 L/min/m^2^ and elevated pulmonary artery wedge pressure ≥ 15 mmHg [2].

Although septic shock and cardiogenic shock have different pathophysiology and hemodynamic profiles, sepsis-induced cardiomyopathy—a new-onset, reversible condition—often occurs alongside sepsis [3], with a reported prevalence of 20% in patients with sepsis [4]. Several mechanisms have been proposed to explain sepsis-induced myocardial dysfunction, including acute changes in loading conditions, myocardial ischemia, and chemical mediators such as pathogen-associated molecular patterns, inflammatory cytokines, nitric oxide, and damage-associated molecular patterns [5]. While septic shock can remain the main pathology of hemodynamic instability even with co-existing cardiac dysfunction, sepsis-induced cardiomyopathy can lead to hypoperfusion. The Surviving Sepsis Campaign Guidelines suggest adding dobutamine to norepinephrine or using epinephrine alone in cases of septic shock with cardiac dysfunction and persistent hypoperfusion, despite adequate fluid resuscitation and stable arterial blood pressure [6]. Unfortunately, the guidelines’ definitions of cardiac dysfunction and hypoperfusion are somewhat ambiguous, leading to varied interpretations among clinicians. In addition, “How to better characterize left ventricular (LV) systolic function?” and “Do we need to treat LV systolic dysfunction?” were included in the research priorities recently published by the Surviving Sepsis Campaign Research Committee [7].

While the Surviving Sepsis Campaign Guidelines suggest the use of inotropes, how to diagnose sepsis-induced cardiogenic shock (SICS) and how to manage SICS remains controversial. In addition, studies regarding SICS are very limited. Hence, further understanding of diagnosis and management of SICS will be important given the aging society and increase in the prevalence of cardiovascular diseases. In this review, we explored the prevalence, diagnosis, and management of SICS.

### Definition of SICS

**Fig. 1:**
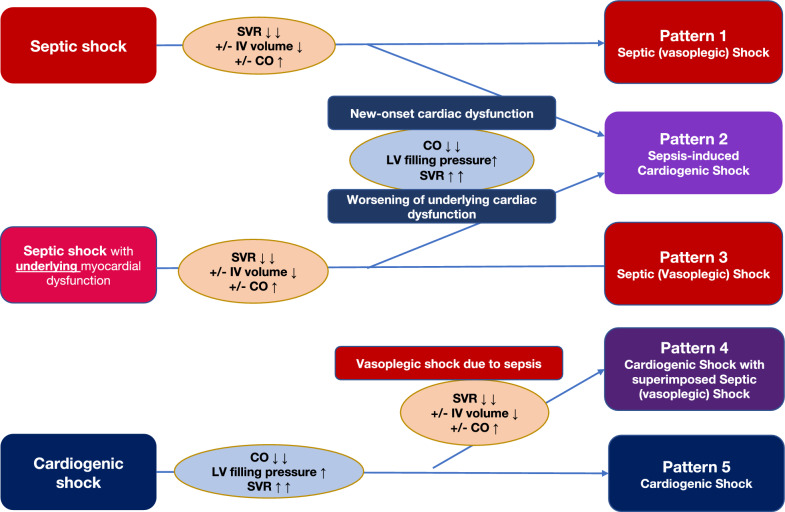
Five patterns of associations between septic shock and cardiogenic shock

SICS can be defined as sepsis complicated by new-onset myocardial dysfunction or worsening of underlying myocardial dysfunction due to sepsis-induced cardiomyopathy, resulting in cardiogenic shock. Differentiating SICS and primary cardiogenic shock complicated by acquired infection can be clinically challenging, as their clinical presentations often overlap and share similar features. The relationship between septic shock and cardiogenic shock can be complex, and five patterns of associations are outlined in Fig. 1. It is important to note that the relationship between vasoplegic shock and cardiogenic shock is continuous in nature and can be difficult to dichotomize in the clinical setting.

### Clinical patterns of septic and cardiogenic shock

#### Pattern 1: septic shock with preserved cardiac function

In this pattern, cardiac output is typically either normal or even increased, while systemic vascular resistance (SVR) is low. This is the classic presentation of septic shock with no significant myocardial dysfunction. Management typically includes antibiotics, intravenous fluid, and vasopressors.

#### Patterns 2: septic shock with new-onset cardiac dysfunction or worsening of pre-existing cardiac dysfunction (SICS)

This pattern represents septic shock complicated by new-onset cardiac dysfunction or the exacerbation of re-existing cardiac dysfunction, leading to SICS. Not all cases of cardiac dysfunction in sepsis will progress to cardiogenic shock, but this group typically presents decreased cardiac output, elevated LV filling pressure, and increased SVR. This is the primary focus of this review.

#### Patterns 3: septic shock with pre-existing cardiac dysfunction (without development of SICS)

In this scenario, patients with chronic cardiac conditions (such as chronic heart failure with reduced ejection fraction) experience septic shock, but do not develop SICS. The primary pathology in this case is vasoplegic shock rather than cardiogenic shock and the underlying cardiac dysfunction does not contribute to hemodynamic instability. Management also involves antibiotics, intravenous fluid, and vasopressors.

#### Pattern 4: primary cardiogenic shock complicated by septic shock (cardiogenic shock with superimposed sepsis)

This pattern involves primary cardiogenic shock that is subsequently complicated by an infectious process leading to septic shock. While many of the principles discussed for SICS may apply here, the pathophysiology and outcomes of patients in this group can differ significantly. Differentiating between the two conditions requires careful assessment of cardiac output, the degree of vasoplegia or vasoconstriction, and loading conditions.

#### Pattern 5: primary cardiogenic shock without superimposed sepsis

In primary cardiogenic shock, cardiogenic output is significantly reduced, and SVR is elevated. This is the classic presentation of cardiogenic shock. Management often includes vasopressors, inotropes, and mechanical circulatory support (MCS).

### Risk factors and prevalence of SICS

While septic shock and cardiogenic shock have been individually studied extensively, the risk factors and prevalence of SICS remain poorly understood. Known risk factors for sepsis-induced cardiomyopathy includes younger age, a history of congestive heart failure, higher lactic acid levels on admission, and positive blood cultures [8, 9]. In contrast, pre-existing cardiac conditions, which are more prevalent in the elderly, are identified as the strongest risk factor for SICS [10]. As the global population ages, the prevalence of cardiac conditions is expected to rise, thereby increasing the significance of SICS in the future. A recent study estimated that SICS, defined as the primary diagnosis of septic shock and secondary diagnosis of cardiogenic shock, occurred in approximately 4.6% of patients with septic shock and was associated with significantly higher mortality compared to septic shock alone [11]. While this study could not confirm the diagnostic criteria for SICS due to the nature of large administrative database, a recent retrospective study using cardiac index ≤ 2.1 L/min/m^2^ revealed the prevalence of 3.7% [10]. Therefore, the prevalence of SICS among septic shock is likely between 3 and 5%. Given sepsis-induced cardiomyopathy develops in about 20% of sepsis cases [4], not all cases of co-existing cardiac dysfunction result in SICS. Rather, only a small proportion of sepsis-induced cardiomyopathy appears to lead to SICS. Nonetheless, SICS remains a significant concern given that septic shock is a very common public health burden and the prevalence is increasing. In addition, a recent study revealed the strongest predictor for developing SICS was a previous history of cardiac dysfunction [10], which has been increasing due to the aging population. Therefore, the prevalence and significance of SICS are expected to increase in the future.

### Diagnosis of SICS

A new-onset reduction in LV ejection fraction to less than 50%, or a decrease of more than 10% from baseline during sepsis has been used in previous retrospective studies to define sepsis-induced cardiomyopathy [8, 9]. However, there is currently no consensus on the diagnostic criteria for this condition. Sepsis-induced myocardial dysfunction can present as left or right, systolic or diastolic dysfunction, complicating the diagnosis further. The prevalence of LV systolic dysfunction, LV diastolic dysfunction, and right ventricular (RV) injury during sepsis is reported to be 20% [4], 48% [12], and 35% [13], respectively. It remains unclear whether sepsis-induced cardiomyopathy itself necessitates deviations from standard of care; however, the presence of cardiogenic shock is likely to require modifications to the management strategy. While the diagnostic criteria for SICS remain undefined, it may be reasonable to apply similar criteria used for cardiogenic shock given that this definition is widely accepted [14]. Cardiogenic shock is generally defined as a state of end-organ hypoperfusion resulting from cardiac dysfunction. Several definitions of cardiogenic shock have been proposed, but the diagnostic criteria typically include persistent hypoperfusion (systolic blood pressure < 90 mmHg) along with a severely reduced cardiac index (< 1.8 L/min/m^2^ without support or < 2.0–2.2 L/min/m^2^ with support), and adequate or elevated filling pressures (LV end-diastolic pressure > 18 mm Hg or a RV end-diastolic pressure > 10–15 mm Hg). A recent retrospective study employed comparable diagnostic criteria (cardiac index ≤ 2.1 L/min/m^2^ in the presence of sepsis-induced cardiac dysfunction) [10]. On the other hand, loading conditions in sepsis can fluctuate dynamically and it is crucial to rule out low cardiac index due to low preload to left ventricle before diagnosing SICS.

The National Cardiogenic Shock initiative, it is recommended to calculate Cardiac Power Output (CPO) and Pulmonary Artery Pulsatility Index (PAPI) for prognostication and treatment guidance during cardiogenic shock [15].

CPO is calculated as

$${\text{CPO}}\, = \,\frac{{{\text{MAP}}\, \times \,{\text{CO }}}}{451}$$

where MAP is mean arterial pressure and CO is cardiac output.

PAPI is calculated as

$${\text{PAPI}}\, = \,\frac{{{\text{PASP }}{-}{\text{ PADP }}}}{{{\text{RAP}}}}$$

where PASP is pulmonary arterial systolic pressure and PADP is pulmonary arterial diastolic pressure, and RAP is right atrial pressure.

CPO is a strong predictor of clinical outcomes in cardiogenic shock, and a CPO value of less than 0.6 may indicate severe cardiogenic shock, while PAPI < 1.0 can help differentiate RV involvement. Therefore, in addition to cardiac index, calculating CPO and PAPI may also aid in diagnosing cardiogenic shock and could be applied to SICS.

**Fig. 2:**
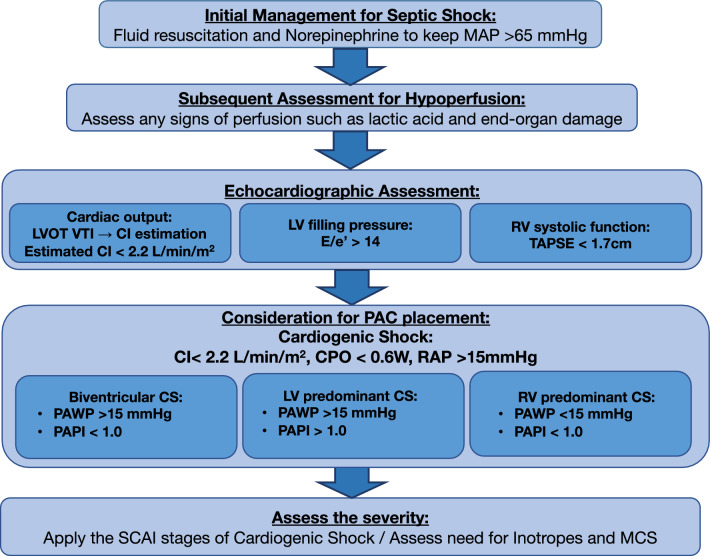
Diagnostic workflow for sepsis-induced cardiogenic shock. *MAP* mean arterial pressure, *LVOT VTI* left ventricular outflow tract velocity time integral, *TAPSE* tricuspid annular plane systolic excursion, *PAC* pulmonary artery catheter, *CI* cardiac index, *CPO* cardiac power output, *PAPI* pulmonary arterial pulsatility index, *SCAI* society for cardiovascular angiography and interventions

Cardiogenic shock is a spectrum rather than a single entity and patients may present with diverse hemodynamic and metabolic abnormalities. To assess the severity and predict outcomes in cardiogenic shock, the Society for Cardiovascular Angiography and Interventions (SCAI) developed a standardized a standardized five-stage classification system. This system categorizes patients from A (at risk of developing cardiogenic shock) and to E (extremis, most severe form of cardiogenic shock). This classification has been validated for predicting mortality in the cardiac intensive care unit and hospital settings [16]. This severity classification may also be applicable to SICS and we recommend to apply the SCAI classification of cardiogenic shock to SICS as well. The suggested diagnostic workflow of SICS is shown in Fig. 2.

#### Echocardiography

Echocardiography is a noninvasive diagnostic modality, which can be performed at the bedside. Echocardiography can roughly estimate cardiac index and pulmonary artery wedge pressure using left ventricular outflow tract velocity integral (LVOT VTI) [17] and E/e’ [18]. The normal value for LVOT VTI is above 18 cm [19]. On the other hand, patients with cardiogenic shock typically present much lower LVOT VTI. A large retrospective study of patients with cardiogenic shock admitted to the cardiac intensive care unit revealed that a LVOT VTI cutoff of 13.2 cm was the single best predictor of hospital mortality among various echocardiographic parameters [20]. Therefore, when LVOT VTI is less than or close to 13.2 cm, it may indicate a particularly high risk of mortality. An E/e’ greater than 14 may indicate elevated LV filling pressure, suggesting possible congestion [21]. In addition, it is well known that sepsis can lead to right ventricular injury [22] and it is associated with worse mortality [23]. While tricuspid annular plane systolic excursion (TAPSE) and the peak tricuspid annular systolic velocity (S’) have been commonly used to assess right ventricular systolic function, it is notable that TAPSE and S’ are both regional systolic measurements, which may not necessarily represent the global function. Right ventricular fraction area change may overcome such a limitation. On the other hand, given the easiness of TAPSE or S’, it may be reasonable to use TAPSE or S’ as a screening for high-risk patients for SICS. Therefore, echocardiography offers a viable screening tool for high-risk patients for SICS. Echocardiography also enables clinicians to profile the type of cardiogenic shock (left, right, or biventricular), which can be extremely helpful to guide the management plan. Even in the absence of a specific suspicion for SICS, a retrospective study has suggested the potential benefit of echocardiography in patients with sepsis [24]. Furthermore, echocardiography can assist in evaluating other differential diagnoses of cardiogenic shock, such as stress-induced (Takotsubo) cardiomyopathy, characterized by regional wall motion abnormalities without coronary artery disease, typically presenting with mid-to-apical hypokinesis and apical ballooning [25]. This can lead to basal hyperkinesis of the left ventricle, potentially causing LVOT obstruction and altering treatment strategies. In our previous study, the prevalence of SICS was very low in patients with sepsis, but became nonnegligible in patients with septic shock [11], suggesting that evaluating cardiac function in all septic patients without clinical signs of shock may not be efficient or practical. Therefore, it may be reasonable to routinely assess cardiac function with echocardiography in patients who require vasopressor support. Measurements of LVOT VTI, E/e’, and TAPSE can help screen for SICS among patients with septic shock.

#### Pulmonary artery catheter

While a previous meta-analysis has not confirmed the overall advantage of pulmonary artery catheter (PAC) in critically ill patients [26], many studies included in this meta-analysis did not specifically address the use of PAC in cardiogenic shock. There is evolving evidence regarding the use of PAC in cardiogenic shock [27, 28], which raises a question of whether SICS can benefit from PAC. Our recent study indicated that patients with SICS might benefit from early invasive hemodynamic monitoring with PAC, whereas those with septic shock alone did not show similar benefits [29]. In addition, this beneficial effect on mortality was limited to early use (within 2 days of admission) of PAC, highlighting that PAC itself is not a treatment but a tool whose impact depends on subsequent action. Therefore, the use of PAC needs to be early enough to meaningfully change management. In addition, myocardial depression often occurs on the first day [30] or even within several hours of presentation [31]. Given echocardiography is available to estimate cardiac index, PAC should be considered if echocardiographic screening raises concerns about cardiogenic shock.

This diagnostic workflow should be standardized, as timely assessment of the need for PAC is imperative. As outlined in *Echocardiography* section, we suggest routinely performing bedside echocardiography in patients requiring vasopressors. When the calculated cardiac index is ≤ 2.2 L/min/m^2^, measuring E/e’ and TAPSE can help determine whether the patient is experiencing LV failure, RV failure or both. In these cases, clinicians may evaluate the necessity of PAC. PAC can help differentiate the primary driver of shock in complex cases. Effective management of SICS requires accurate volume assessments, adequate selection of vasoactive and inotropic agents, and their goal-directed titration, all of which can be guided by PAC. According to the National Cardiogenic Shock Initiative protocol evaluated across 80 hospitals in the United States, maintaining CPO > 0.6 and PAPI > 0.9 is recommended [15]. Although this protocol was designed for cardiogenic shock due to acute myocardial infarction, it could also help determine whether patients with SICS need escalation to mechanical circulatory support (MCS). Our study reported that MCS was more frequently utilized in patients with SICS who had undergone PAC monitoring [29]. Since MCS is less effective in vasoplegic shock, or can even be contraindicated, it is essential to identify the predominant driver of shock accurately. On the other hand, routine PAC monitoring for all septic patients is impractical, and the associated risks must be considered. Previous clinical trials suggested the PAC complication rates of 5–10% [32, 33], with most complications being non-fatal, though pulmonary artery rupture, which can be fatal, has an incidence of approximately 0.03% [34]. Therefore, clinicians must carefully evaluate the potential benefits of PAC for individual patients and address specific clinical questions that PAC can help answer.

### Management of SICS

#### Pharmacologic approach

The impact of inotropes, as suggested by SSCG for suspected SICS, on mortality remains uncertain. While SSCG suggested adding dobutamine to norepinephrine or the use of epinephrine alone in SICS, this suggestion was based on the results from the network meta-analysis by Belletti et al. [35]. However, there were no significant difference in mortality among norepinephrine, norepinephrine plus dobutamine, and epinephrine in this network meta-analysis, suggesting that its results do not necessarily endorse the recommendation by SSCG. Our prior study indicated a potential worsening of outcomes with inotrope use in septic shock patients [36]. Even in patients with severe heart failure, a meta-analysis showed no mortality benefit with the use of dobutamine [37]. In addition, a propensity-score matched retrospective study using the Acute Decompensated Heart Failure National Registry showed the use of inotropes doubled in-hospital mortality [38]. Furthermore, a previous clinical trial revealed that the use of dopamine, which is an inotropic agent, increased a risk of death compared to norepinephrine in cardiogenic shock [39]. Another randomized controlled trial showed epinephrine was associated with more refractory shock than norepinephrine [40]. Finally, an individual participant data meta-analysis showed that epinephrine increased risk of death to threefold in cardiogenic shock [41]. All these studies suggest against inotropes. On the other hand, many studies included cardiogenic shock due to acute myocardial infarction and an increased oxygen demand due to inotropes may explain these negative findings. This raises the question of whether a non-catecholaminergic inotropic agent is preferable to catecholaminergic options. On the other hand, a randomized controlled trial in patients with cardiogenic shock, the majority of whom had ischemic etiology, found no significant clinical differences between dobutamine and milrinone [42]. Therefore, there is no definitive evidence supporting the superiority of one over the other.

On the other hand, despite the lack of supporting evidence, it remains reasonable to use inotropes to increase cardiac output when reduced cardiac output is felt to impair oxygen delivery. Once clinicians determine to initiate inotropes—aimed at improving tissue oxygen delivery by increasing cardiac output—monitoring the response to inotropes, such as reduction in lactic acid levels, is essential. It is advisable to maintain a cardiac index greater than 2.2 L/min/m^2^ in patients with SICS. A pilot randomized trial showed the use of dobutamine to optimize ventriculo-arterial coupling in septic shock improved lactic acid clearance and had a tendency of lower in-hospital mortality [43]. While this is a relatively small pilot trial, it may suggest that the manner in which inotropes are titrated can affect outcomes. Nevertheless, given the lack of concrete evidence for inotropes, clinicians should exercise caution in their use.

For severe RV injury accompanied by elevated pulmonary artery pressure, pulmonary vasodilators such as inhaled nitric oxide or epoprostenol may provide temporary RV support by reducing afterload. These agents have been used as rescue therapy at the discretion of clinicians. However, there is a significant lack of studies investigating the use of pulmonary vasodilators in the context of RV injury during sepsis.

#### Mechanical circulatory support (MCS)

It is important to note that cardiogenic shock differs from septic shock by limited evidence for pharmacological support and the availability of MCS. When cardiogenic shock is the predominant component over vasoplegic shock due to sepsis, MCS may be beneficial as it enhances cardiac output. Early initiation of MCS, particularly in severe cases where patients require more than one inotropic agent or high-dose vasopressors to maintain systemic circulation, is worth exploring, since the use of high-dose vasopressors and inotropes is associated with worse outcomes [44]. Given that sepsis-induced cardiomyopathy is a reversible condition, MCS may serve as a bridge to recovery. On the other hand, high-quality studies regarding the use of MCS in the setting of SICS remain critically lacking.

When MCS is considered for SICS, a strategy similar to that used for cardiogenic shock due to acute myocardial infarction can be applied. For instance, if CPO remains below 0.6 despite inotropic support, escalating to MCS may be warranted. If PAPI is ≤ 0.9, right-sided MCS may be considered. While intra-aortic balloon pump (IABP) and peripheral left ventricular assist device (pLVAD) such as Impella CP/5.0/5.5 are often utilized to support LV in cardiogenic shock and less invasive than extracorporeal membranous support (ECMO), there have been only case reports and case series for the use of IABP and pLVAD in SICS [45, 46] except for a recent large administrative database study [47]. This recent study investigated the use of MCS in patients with SICS with and without acute myocardial infarction (AMI) as acute infection is a known risk factor for acute myocardial infarction. Interestingly, IABP and pLVAD were not associated with lower mortality in SICS complicated by AMI, while they were in SICS without AMI, suggesting that temporary MCS could be a viable option for those with SICS due to sepsis-induced cardiomyopathy [47]. In this study, the average timing of MCS initiation was 48–72 h from admission, which likely reflects a trial of standard hemodynamic management for sepsis during the first 24–48 h. Applicability of this study should be interpreted cautiously as it was not designed to support causal inference, and may contain hidden confounders due to the nature of the large administrative database. While this study did not show any benefit with ECMO, the use of veno-arterial ECMO (VA ECMO) has been studied more than other MCS in SICS. While a systematic review suggested very high mortality in septic patients who underwent VA ECMO [48], a propensity score matched multicenter retrospective study showed mortality benefit with VA ECMO in patients with SICS [49]. In this study, mean cardiac index for patients who underwent VA ECMO was 1.5 L/min/m^2^, which indicates severe myocardial dysfunction. The biggest difference between previous studies that showed higher mortality and this study appeared to be the presence of refractory cardiogenic shock. In fact, a meta-regression analysis found better outcomes in SICS patients with lower LV systolic function who underwent VA ECMO, suggesting the importance of proper patient selection for MCS [50].

#### Cardiogenic shock team

**Fig. 3:**
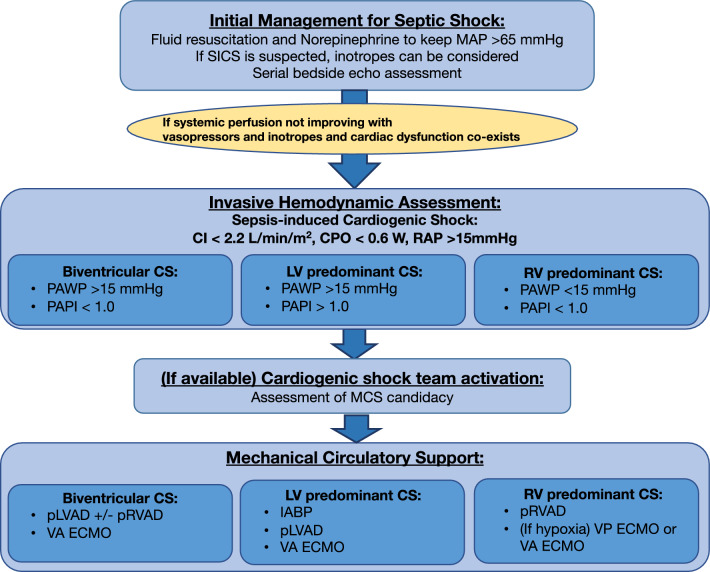
Therapeutic workflow for sepsis-induced cardiogenic shock. *MAP* mean arterial pressure, *CI* cardiac index, *CPO* cardiac power output, *IABP* intra-aortic balloon pump, *pLVAD* percutaneous left ventricular assist device, *pRVAD* percutaneous right ventricular assist device, *VA ECMO* venoarterial extracorporeal membranous oxygenation, *VP ECMO* veno-pulmonary artery extracorporeal membranous oxygenation

Engaging a cardiogenic shock team—if available—for a multidisciplinary approach involving critical care, cardiology, advanced heart failure cardiology, interventional cardiology, and cardiac surgery can be beneficial [51]. The concept of cardiogenic shock team originated from the Detroit Cardiogenic Shock Initiative in 2018, which involved four hospitals in Detroit, Michigan, focusing on the early use of MCS in acute myocardial infarction complicated by cardiogenic shock [52]. Building on this, in 2023, the National Cardiogenic Shock Initiative, published in 2023 and encompassing 80 hospitals across the United States, evaluated the feasibility and impact of implementing a shock protocol for acute myocardial infarction-induced cardiogenic shock with a multidisciplinary team approach [15]. This shock protocol includes the diagnostic criteria and practice recommendations, with an emphasis on the early use of mechanical circulatory support. The time sensitive nature of cardiogenic shock and the need for coordinated inputs from multiple stakeholders often necessitate a team-based approach. This is particularly important in SICS, where the complexity of hemodynamics, due to the co-existence of vasoplegia and the unique risks posed by infection, emphasizes the need for simultaneous contributions from various experts. The suggested therapeutic workflow was shown in Fig. 3.

## Conclusions

This article highlights the need to address the evidence gap in diagnosing and managing SICS. Echocardiography for screening, advanced monitoring with PAC, and consideration for MCS candidacy should be prioritized for patients with SICS.

## Data Availability

No datasets were generated or analyzed.

## Abbreviations

SSCG: The Surviving Sepsis Campaign Guidelines
SICS: Sepsis-induced cardiogenic shock
PAC: Pulmonary artery catheter
PAPI: Pulmonary artery pulsatility index
CPO: Cardiac power output
LVOT VTI: Left ventricular outflow tract velocity integral
TAPSE: Tricuspid annular plane systolic excursion
MCS: Mechanical circulatory support
IABP: Intra-aortic balloon pump
pLVAD: Peripheral left ventricular assist device
AMI: Acute myocardial infarction
VA ECMO: Venoarterial extracorporeal membrane oxygenation
